# ADA-HF: acetazolamide as a chloride sparing diuretic in patients admitted to hospital with heart failure

**DOI:** 10.1007/s00392-026-02915-5

**Published:** 2026-04-27

**Authors:** J. J. Cuthbert, E. Luo, A. S. M. Ahmed, M. Ajith, H. Butt, H. Pinhol, F. Baffour Korsah, J. Bulemfu, S. Ford, G. Constable, L. Cox, A. S. Rigby, A. L. Clark

**Affiliations:** 1 Wolfson Palliative Care Research Group, Hull York Medical School, University of Hull, Cottingham Road, Kingston-Upon-Hull, Hull, HU6 7RX UK; 2 Department of Cardiology, Castle Hill Hospital, Hull University Teaching Hospital NHS Trust, Castle Road, Cottingham, Kingston-Upon-Hull, HU16 5JQ UK; 3 Research and Development, Castle Hill Hospital, Hull University Teaching Hospital NHS Trust, Castle Road, Cottingham,, Kingston-Upon-Hull, HU16 5JQ UK; 4 Hull York Medical School, University of Hull, Cottingham Road, Kingston-Upon-Hull, Cottingham, HU6 7RX UK

**Keywords:** Diuretics, Heart failure, Acetazolamide, Hospitalisation, Venous congestion, Furosemide

## Abstract

**Background:**

We assessed whether oral acetazolamide (ACZ) increases diuresis and reduces chloride loss when given alongside high-dose intravenous (IV) furosemide in patients admitted to hospital with HF.

**Methods and Results:**

ADA-HF was a single-centre, open-label, randomised controlled trial. Patients were randomised to ACZ 250 mg twice daily plus high-dose (240 mg per day) IV furosemide infusion (standard of care (SoC)) versus SoC alone for 4 days. The co-primary endpoints were (1) daily net fluid loss between baseline and day 4 and (2) change in serum chloride level from baseline to day 4. A total of 46 patients (median age, 76; 65% male; median N-terminal pro-B-type natriuretic peptide, 4097 ng/L) from a screened population of 207 were randomised (23 to ACZ, 23 to SoC). The median daily net fluid loss was 1073 mL (1st–3rd quartile range 682–1419 mL) in the ACZ arm vs. 1029 mL (201–1432 mL) in the SoC arm (*P* = 0.51). There was no change in serum chloride concentration in the ACZ arm, whereas chloride fell by 7 (2–10) mmol/L in the SoC arm (*P* < 0.001). The number of adverse and serious adverse events ((S)AE) was numerically greater in the ACZ arm (51 events in 16 patients vs. 29 events in 10 patients, *P* = 0.24). Overall, 8 patients (30%) experienced (S)AEs possibly related to ACZ, and two of whom withdrew from the trial.

**Conclusions:**

Acetazolamide has a significant “chloride-sparing” effect in patients given high-dose IV furosemide. However, ACZ did not significantly increase diuresis, and side effects from oral ACZ were frequent.

**Trial registration.:**

ISRCTN13060336; 9/2/2023.

**Graphical Abstract:**

**A** change in fluid balance and **B** change in serum chloride; HF, heart failure; ACZ, acetazolamide; SoC, standard of care; RCT, randomised controlled trial; NT-pro-BNP, N-terminal pro-B-type natriuretic peptide

**Figure:**
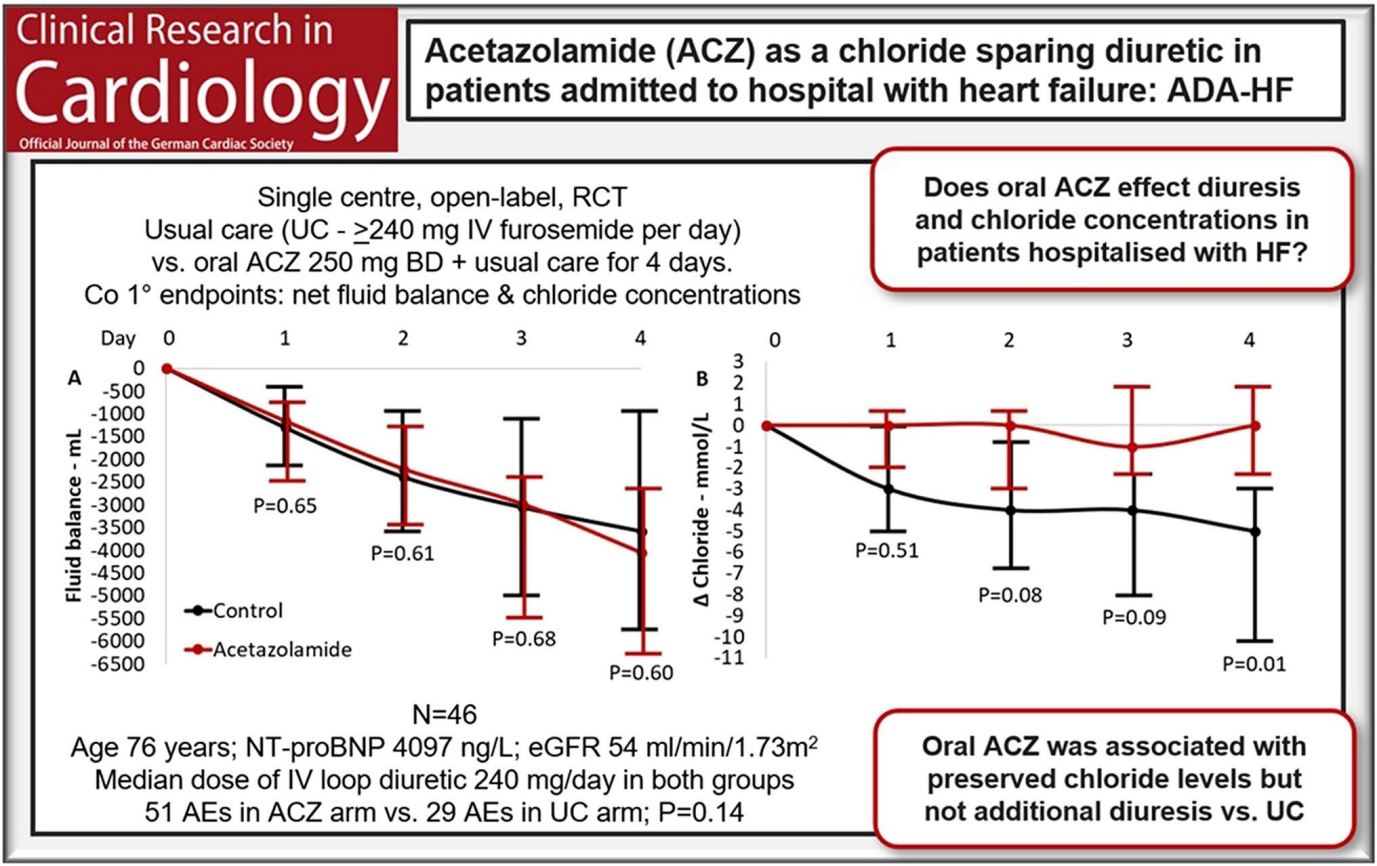

**Supplementary Information:**

The online version contains supplementary material available at 10.1007/s00392-026-02915-5.

## Introduction

Hypochloraemia is a common complication of loop diuretic treatment in patients with heart failure (HF), which is associated with increased neurohormonal activation, diuretic resistance, and an increased risk of adverse outcomes [1, 2]. Whether hypochloraemia is a marker or mediator of adverse outcome is unclear. There are few strategies to prevent or treat hypochloraemia, but acetazolamide has been mooted [3].

There have been several trials of acetazolamide in patients hospitalised with HF. The largest is ADVOR, which found a modest increase in diuresis with acetazolamide when given with low-dose IV furosemide compared to furosemide alone (~ 120 mg per day) [4–7]. Post hoc analysis of the ADVOR trial also found that acetazolamide may have a “chloride sparing” effect [8].

However, because ADVOR used IV acetazolamide, and a placebo alongside a low-dose loop diuretic as the comparator, whether acetazolamide has a chloride-sparing diuretic effect when given orally alongside high-dose IV furosemide in patients hospitalised with HF is unknown. We designed the ADA-HF (acetazolamide as a chloride-sparing diuretic in patients admitted to hospitals with HF) pilot study to test the hypothesis that oral acetazolamide would reduce urinary chloride loss and increase diuresis in patients hospitalised with HF who were receiving high-dose IV furosemide, compared to furosemide alone.

## Methods

### Study design

ADA-HF was a single-centre, open-label, randomised controlled trial designed and conducted by the Department of Academic Cardiology at the Hull York Medical School, sponsored by the Hull University Teaching Hospitals NHS Trust in Kingston-Upon-Hull in the UK, and funded by the British Heart Foundation Clinical Research Collaborative Research Development Fund.

The study protocol and statistical analysis plan are included in the supplement, and the study design is described in detail elsewhere [9]. The study was approved by the local Research Ethics Committee (22/EM/0264) and the Health Research Authority, and was performed according to the Medicines for Human Use (Clinical Trials) Regulations and the Declaration of Helsinki. All patients provided written informed consent. The trial is registered with the ISRCTN registry (13,060,336).

### Participants

Adult patients hospitalised with venous congestion due to HF as the primary reason for admission and who were deemed by their clinical team to require high-dose IV furosemide treatment (at least 240 mg IV furosemide per day) were eligible. Patients could be recruited at any point during the hospitalisation, as long as they were deemed to require at least 240 mg of IV furosemide per day. Entry criteria were intentionally broad to enable recruitment of a representative population of patients hospitalised with HF. The titration of IV loop-diuretic dosing was at the discretion of the treating clinician.

Patients were recruited regardless of ejection fraction, HF aetiology, or prior treatment with an oral or IV loop diuretic [10]. Exclusion criteria included but were not limited to: not likely to need IV treatment for at least 4 days, taking a sodium glucose co-transporter 2 inhibitor (SGLT2I) or thiazide (or thiazide-like) diuretic on admission (to ensure the only diuretic treatments given during the study period were acetazolamide and furosemide), or an estimated glomerular filtration rate (eGFR) ≤ 20 mL/min/1.73 m^2^ at the time of screening. The full list of inclusion and exclusion criteria is described elsewhere [9].

### Randomisation and treatment allocation

Patients were randomised 1:1 to receive either acetazolamide 250 mg twice daily for 4 days, or standard of care, using a random permuted block of up to 50 patients using a simple web-based system.

All patients were on a background continuous IV infusion of furosemide at 10 mg/h. If a patient was enrolled before receiving any IV furosemide during hospitalisation the infusion would be given after a bolus of 80 mg IV furosemide, as per usual care. The dose of furosemide could be titrated by the clinical team.

### Follow up and endpoints

All participants had daily in-person follow-up for 4 days (96 h) alongside usual care with telephone consultations after 30 days, and remote follow-up using the electronic health record at 180 days.

The co-primary endpoints were: the difference in daily net fluid loss (fluid intake–urine output), and the difference in change in serum chloride concentration from baseline to day 4 between the two treatment arms.

Secondary endpoints included but were not limited to: change in body weight, change in serum electrolytes, change in eGFR, change in clinical congestion score using the Hull1 and the modified-ADVOR congestion scores [11], change in the inferior vena cava (IVC) diameter, and change in patient-reported symptoms at rest and on exertion using a 5-point Likert score (5, most severe breathlessness; 0, not breathless) between baseline and day 4. A 24-h urine electrolyte excretion was measured between days 1 and 2 and between days 3 and 4. All endpoints were measured by unblinded investigators.

### Sample size and statistical analysis

To guide recruitment for our pilot study, we performed a power calculation to estimate the minimum number of participants required to detect a signal of possible benefit. We estimated that a sample size of 35 patients (18 in the ACZ arm, 17 in the SoC arm) with an expected drop-out rate of 20% in the ACZ arm and 10% in the SoC arm would enable us to detect a 1-mmol/L difference in serum chloride concentration per day with 80% power and a two-tailed level of significance set at 5% [12].

All continuous data are presented as the median (1st and 3rd quartiles), and all categorical data are presented as the number (percentage). All endpoints were assessed in an intention-to-treat analysis. The co-primary endpoints were normally distributed and were assessed using independent samples *t*-tests. Comparisons between all other continuous outcomes were made using independent samples *t*-tests for continuous data, if normally distributed, and Mann–Whitney *U* tests, if non-normally distributed. Chi-squared tests were used for categorical data.

Hospitalisation and mortality data were assessed using univariable Cox regression analysis except for the proportion of patients who were euvolaemic at discharge which was assessed using an odds ratio. All analyses were performed on SPSS version 28. We performed both intention-to-treat and per-protocol analyses. The last patient’s last visit was on 08/07/2024, and database lock occurred on 24/09/2024.

### Patient and public involvement

Patients with HF and their carers were involved in the design of the trial via the INVOLVE Hull patient and public involvement group.

## Results

Between 14/03/2023 and 31/01/2024, 207 patients admitted to Hull University Teaching Hospitals NHS Trust with a primary diagnosis of HF were screened. The most common reason for exclusion was that patients were not expected to require the standard of care for 4 or more days (21%), or were taking an SGLT2I on admission (20%). A total of 46 patients were randomised (23 to acetazolamide, 23 to standard of care). One patient died between screening and randomisation and on the first dose of acetazolamide due to sudden cardiac death. Of the remaining 45 patients, 39 completed the trial: of the 22 assigned to acetazolamide, 3 patients withdrew due to side effects of treatment, and one patient achieved euvolaemia before day 4; of the 23 patients assigned to standard of care, 2 patients achieved euvolaemia before day 4 (Supplementary Fig. 1).

### Patient characteristics

The median age was 76 years, the majority were male, and most were in atrial fibrillation. The median left ventricular ejection fraction (LVEF) was 46%; 54% of patients had an LVEF < 50%. The median NT-proBNP was 4097 ng/L and the median eGFR was 54 mL/min/1.73 m^2^. All patients had peripheral oedema, and most had a raised jugular venous pulse, lung crackles, and pleural effusions. Approximately one in three had ascites. Most patients were taking a loop diuretic prior to admission, with a median daily dose of 40 mg furosemide equivalents. The median duration of admission before randomisation was 2 days, during which time the median cumulative dose of IV furosemide given was 180 mg (Table 1).

**Table 1:** Baseline characteristics

	Missing	All (*N* = 46)	Control (*N* = 23)	Acetazolamide (*N* = 23)
**Demographics**				
Age, years	0	76 (70–81)	77 (69–85)	74 (70–79)
Sex (male), *N* (%)		30 (65)	16 (70)	14 (61)
Height, m	3	1.71 (1.62–1.79)	1.71 (1.65–1.78)	1.69 (1.60–1.80)
Weight, kg	3	91.4 (80.0–107.0)	87.9 (78.2–97.5)	94.9 (80.8–115.0)
Systolic blood pressure, mmHg	0	118 (105–134)	121 (106–145)	116 (103–130)
Heart rate, bpm		75 (64–84)	75 (65–82)	78 (63–89)
Sinus rhythm, *N* (%)		12 (26)	9 (39)	3 (13)
**Comorbidities**				
Ischaemic heart disease, *N* (%)	0	18 (39)	9 (39)	9 (39)
Hypertension, *N* (%)		33 (72)	17 (74)	16 (70)
Diabetes, *N* (%)		18 (39)	8 (35)	10 (44)
COPD, *N* (%)		9 (20)	5 (22)	4 (17)
eGFR < 60 mL/min/1.72m^2^ a, *N* (%)	3	26 (59)	13 (57)	13 (62)
CVA or TIA, *N* (%)	0	4 (9)	1 (4)	3 (13)
Anaemia, *N* (%)		26 (65)	14 (67)	12 (63)
**Heart failure**				
LVEF, %		46 (33–55)	46 (33–55)	45 (33–55)
LVEF < 50%, *N* (%)	0	24 (54)	10 (43)	14 (60)
Valve disease, *N* (%)		3 (7)	2 (9)	1 (4)
IVC diameter, cm	6	2.3 (2.1–2.5)	2.2 (2.1–2.5)	2.4 (2.1–2.8)
IVC diameter > 2.0 cm, *N* (%)		35 (88)	17 (81)	18 (95)
**Physical examination and symptoms**				
Peripheral oedema, *N* (%)	2	44 (100)	23 (100)	21 (100)
Lung crackles, *N* (%)		37 (84)	18 (78)	19 (83)
Pleural effusion, *N* (%)		25 (57)	13 (57)	12 (57)
Raised JVP, *N* (%)		41 (93)	23 (100)	18 (78)
Ascites, *N* (%)		15 (34)	5 (22)	10 (48)
3rd heart sound, *N* (%)		2 (5)	1 (4)	1 (5)
Hull congestion score		6 (4–6)	5 (4–6)	6 (5–7)
Modified ADVOR congestion score		6 (4–7)	5 (4–6)	6 (5–9)
Likert score at rest		1 (1–2)	1 (1–2)	1 (1–3)
Likert score on exertion		4 (4–5)	4 (4–5)	5 (4–5)
**Blood results**				
NTproBNP, ng/L	1	4097 (2240–9207)	3829 (1964–9829)	4154 (2612–9639)
Sodium, mmol/L	3	138 (136–141)	138 (135–143)	138 (137–141)
*Hyponatraemia*^b^, %		6 (14)	4 (18)	2 (10)
Chloride, mmol/L		97 (94–101)	98 (94–103)	95 (94–100)
*Hypochloraemia*^c^, %		20 (47)	9 (41)	11 (52)
Bicarbonate, mmol/L		30 (25–33)	30 (25–32)	31 (26–33)
Potassium, mmol/L		3.8 (3.6–4.3)	4.2 (3.6–4.5)	3.8 (3.4–4.2)
Urea, mmol/L		7.7 (6.2–13.5)	7.9 (6.4–14.5)	7.7 (6.1–11.6)
Creatinine, µmol/L		107 (90–140)	110 (86–143)	104 (95–143)
eGFR, mL/min/1.72m^2a^		54 (39–67)	54 (41–65)	51 (39–67)
Albumin, g/L		32 (30–35)	32 (31–37)	33 (30–34)
Haemoglobin, g/L		118 (105–139)	116 (101–139)	118 (106–139)
Haematocrit, fraction		0.368 (0.312–0.422)	0.343 (0.300–0.396)	0.3789 (0.313–0.448)
**Admission chest X-ray**				
Congestion, %	0	42 (91)	21 (91)	21 (91)
Pleural effusions, %		33 (72)	16 (70)	17 (74)
Pulmonary oedema, %		14 (30)	6 (26)	8 (35)
**Pre-admission medications**				
ACEI/ARB/ARNI, %	0	21 (46)	11 (48)	10 (44)
Beta-blocker, %		35 (76)	17 (74)	18 (78)
MRA, %		8 (17)	5 (22)	3 (13)
Digoxin, %		5 (11)	3 (13)	2 (9)
Loop diuretic, %		36 (78)	19 (83)	17 (74)
Daily dose, mg^e^	10	40 (20–80)	40 (20–80)	40 (0–80)
ICD, %	0	2 (4)	2 (9)	0
CRT, %		0	0	0
Days between admission and randomisation	0	2 (1–4)	2 (1–4)	2 (1–4)
Cumulative dose of IV furosemide between admission and randomisation, mg		180 (80–383)	180 (90–340)	240 (60–500)

*COPD* chronic obstructive pulmonary disease, *eGFR* estimated glomerular filtration rate, *CVA* cerebrovascular accident, *TIA* transient ischaemic attack, *LVEF* left ventricular ejection fraction, *IVC* inferior vena cava, *JVP* jugular venous pulse, *NTproBNP* N-terminal B-type natriuretic peptide, *ACEI* angiotensin converting enzyme inhibitor, *ARB* angiotensin receptor blocker, *ARNI* angiotensin receptor neprilysin inhibitor, *MRA* mineralocorticoid receptor antagonist, *ICD* implantable cardioverter defibrillator, *CRT* cardiac resynchronisation therapy, *IV* intravenous.

^a^Calculated using the CKD-EPI 2021 formula.

^b^ < 135 mmol/L.

^c^ < 96 mmol/L.

^d^ < 3.5 mmol/L.

^e^In furosemide equivalents (1 mg bumetanide = 40 mg furosemide).

*IVC* inferior vena cava.

### Co-primary endpoints

**Fig. 1:**
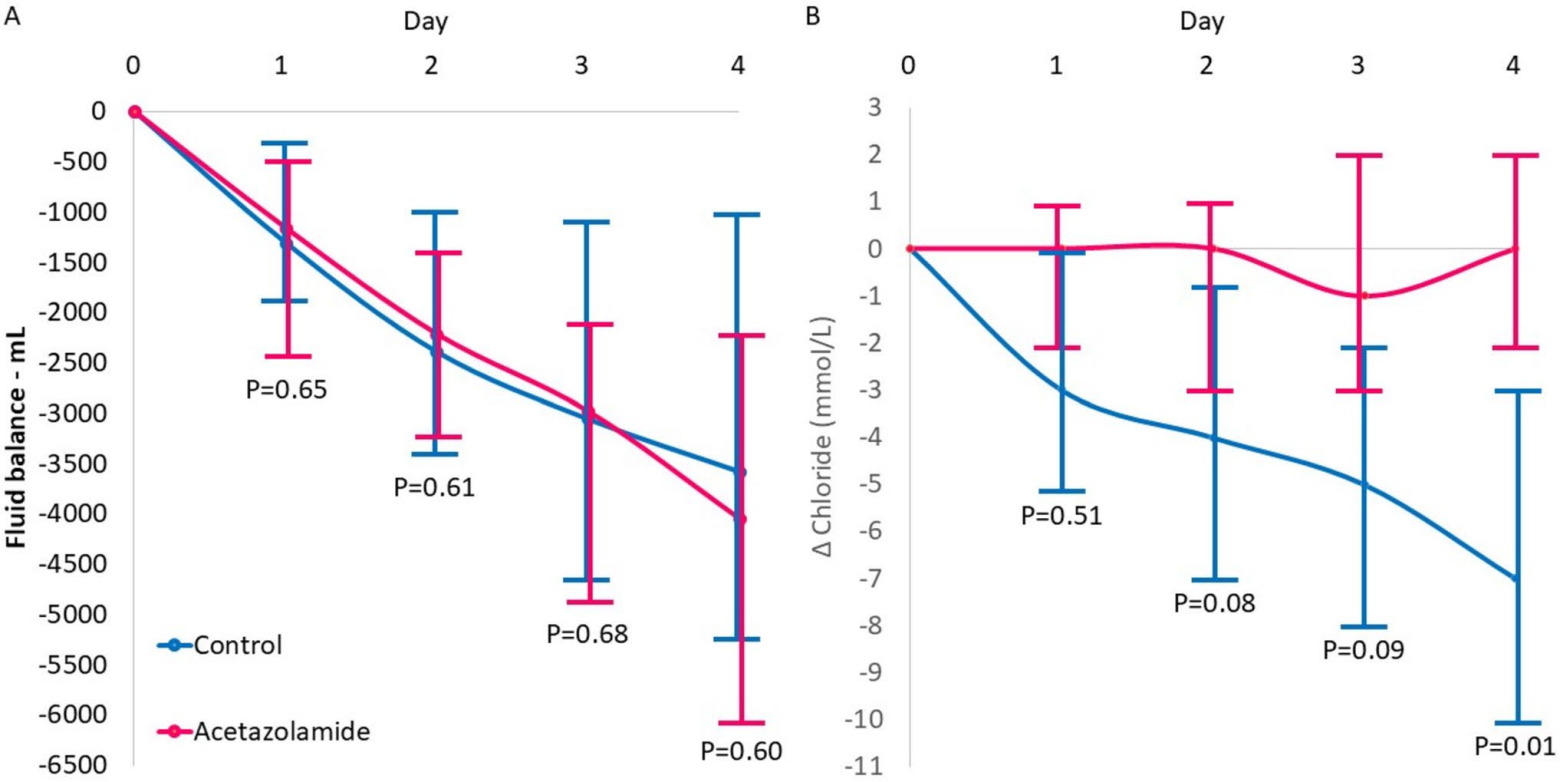
Primary endpoints: **A** net fluid balance and **B** change in chloride concentration from baseline

There was no difference in daily (1029 (201–1432) vs. 1073 (682–1419), *P* = 0.51) or cumulative (3578 (1219–5458) vs. 4070 (2478–6074), *P* = 0.60) net fluid loss between the standard of care and acetazolamide arms after 4 days of treatment. Serum chloride fell by 7 mmol/L in the standard-of-care arm but was unchanged in the acetazolamide arm (*P* < 0.001) (Table 2, Fig. 1, Graphic Abstract). The median daily dose and the median cumulative dose of IV furosemide between baseline and day 4 were similar between the arms (Table 2) and there was no dose titration in either arm during the study period.

**Table 2:** Primary endpoints

	*N*	Control	*N*	Acetazolamide	Between-group difference (ACZ vs. control)	***P***
Daily furosemide dose, mg		240 (236 to 240)		240 (238 to 240)	0	-
Cumulative furosemide dose (96 h), mg		960 (948 to 960)		955 (940 to 960)	5	-
Cumulative fluid balance (mL)						
Baseline, 0 h	23	-	22	-	-	-
Visit 5, 96 h	21	− 3578 (− 5458 to − 1219)	18	− 4070 (− 6074 to − 2478)	− 492	0.60
*Median daily fluid balance (24*–*96 h)*	21	− 1029 (− 1432 to − 201)	18	− 1073 (− 1419 to − 682)	− 44	0.51
Serum chloride (mmol/L)						
Baseline, 0 h	23	98 (94 to 103)	22	95 (94 to 100)	3	0.42
Visit 5, 96 h	21	91 (88 to 97)	18	95 (92 to 100)	4	0.01
*Change in chloride 24*–*96 h*	21	− 7 (− 10 to − 3)	18	0 (− 2 to 2)	7	< 0.001

### Secondary endpoints

**Fig. 2:**
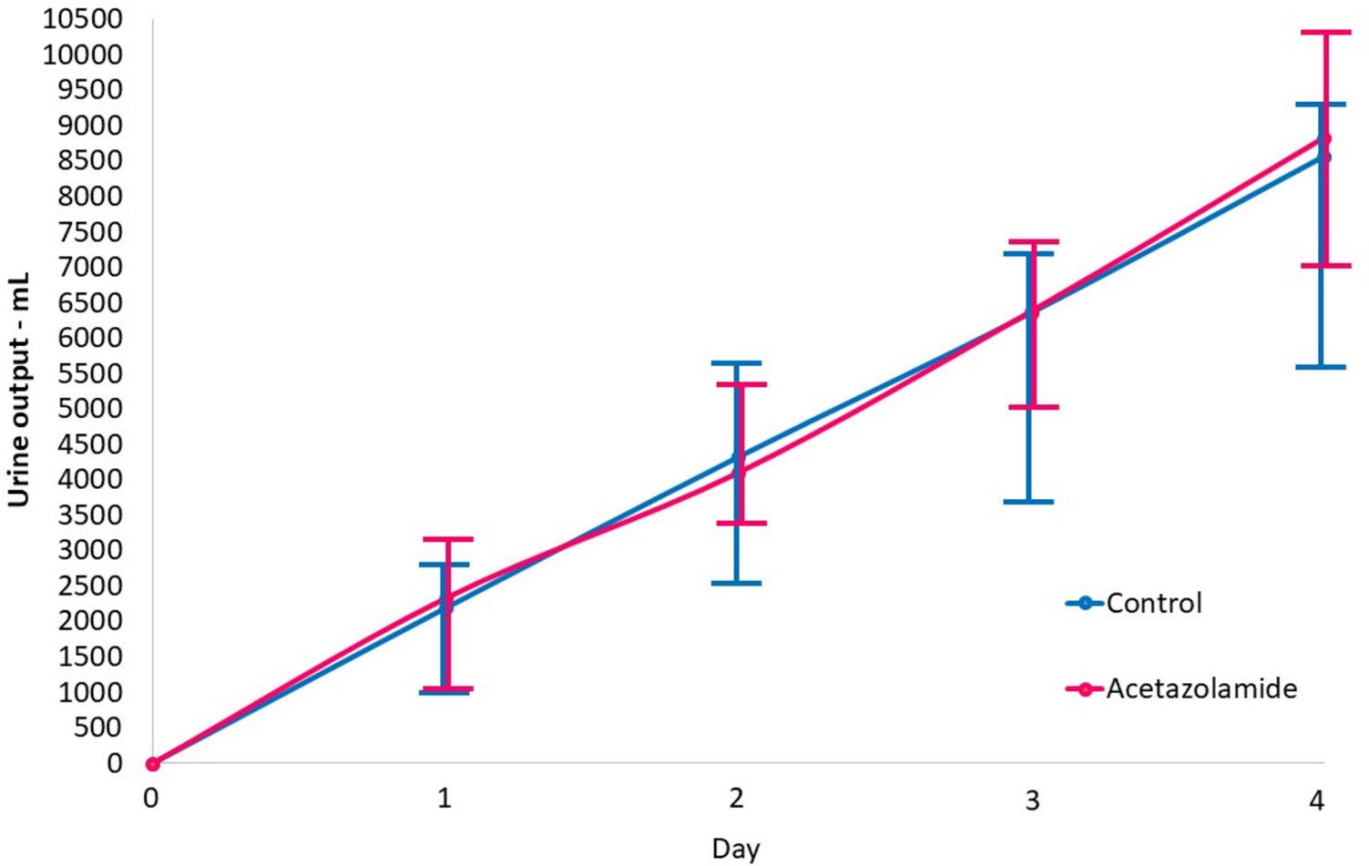
Urine output

There was no difference in urine output (Fig. 2), weight loss, change in congestion scores, or IVC diameter between the arms. However, patients in the acetazolamide arm had greater improvement in patient-reported breathlessness on exertion (Table 3) (*P* = 0.01).

**Table 3:** Secondary and exploratory endpoints

	*N*	Control	*N*	Acetazolamide	Between-group difference ACZ vs. control	***P***
Daily furosemide dose, mg		240 (236 to 240)		240 (238 to 240)	−	−
Cumulative furosemide dose (96 h), mg		960 (948 to 960)		955 (940 to 960)	5	−
Urine output (mL)						
Baseline, 0 h	23	0	22	0	0	−
Visit 5, 96 h	21	8560 (5854 to 9275)	18	8820 (7164 to 10211)	260	0.51
*Median daily urine output (24–96 h)*	21	1888 (1465 to 2235)	18	2258 (1569 to 2698)	370	0.31
Change in weight (kg)						
Baseline, 0 h	23	0	22	0		
Visit 5, 96 h	21	− 5.6 (− 7.7 to − 1.5)	18	− 3.7 (− 6.6 to − 2.5)	1.6	0.82
Change in the Hull congestion score						
Baseline, 0 h	23	5 (4 to 6)	22	6 (5 to 7)	−	−
Visit 5, 96 h	21	− 3 (− 5 to − 2)		− 3 (− 5 to − 2)	0	0.48
Change in the modified ADVOR congestion score						
Baseline, 0 h	23	5 (4 to 6)	22	6 (5 to 9)	−	−
Visit 5, 96 h	21	− 3 (− 4 to − 1)	18	− 4 (− 3 to − 6)		0.21
Change in Likert score at rest						
Baseline, 0 h	23	1 (1 to 2)	22	1 (1 to 3)	−	−
Visit 5, 96 h	21	0 (− 1 to 0)	18	− 1 (− 1 to 0)	− 1	0.13
Change in Likert score on exertion						
Baseline, 0 h	23	4 (4 to 5)	22	5 (4 to 5)	−	−
Visit 5, 96 h	21	− 1 (− 2 to 0)	18	− 2 (− 2 to − 1)	− 1	0.01
Change in IVC diameter (cm)						
Baseline	21	2.2 (2.1 to 2.5)	19	2.4 (2.1 to 2.8)	−	−
Visit 5, 96 h	16	− 0.2 − 0.5 to − 0.1)	12	− 0.4 (− 0.6 to − 0.2)	− 0.2	0.63
IVC diameter > 2.0 cm at baseline, *N* (%)	21	17 (81)	19	18 (95)	−	−
IVC diameter > 2.0 cm at 96 h, *N* (%)	16	7 (41)	12	7 (54)	−	0.37

### Urinary electrolytes

**Fig. 3:**
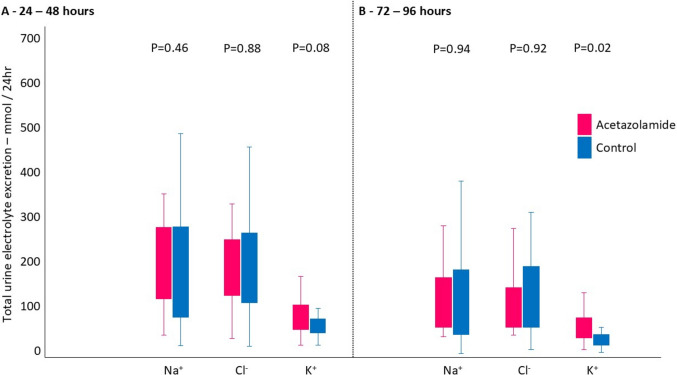
Urinary electrolytes: **A** 24–48 h, *N* of assessed ACZ = 19 and SoC = 21, **B** 72–96 h, *N* of assessed ACZ = 11 and SoC = 11

Between days 1 and 2 (24–48 h), there was no difference in urinary concentrations or total urinary excretions of sodium, chloride, or potassium. Between days 3 and 4 (76–96 h), the total potassium excretion was greater in the acetazolamide arm compared to the standard of care, but there was no difference in the urine sodium or chloride concentrations or total excretion (Supplementary Table 2) (Fig. 3).

### Safety endpoints

There was no difference in safety endpoints or adverse events between the two arms (Supplementary Tables 2 and 3). However, adverse events, notably hypotension and hypokalaemia, were numerically greater in the acetazolamide arm than the standard-of-care arm (51 events in 16 patients vs. 29 events in 10 patients, *P* = 0.14). There was no difference in the rates of hyponatraemia or worsening renal function. Although serum bicarbonate fell by 3 mmol/L in the acetazolamide arm and rose by 4 mmol/L in the standard-of-care arm (*P* = 0.05), there was no difference in the rates of metabolic acidosis or alkalosis (Supplementary Table 2).

Approximately one in three patients in the ACZ arm reported side effects that are not commonly attributed to furosemide treatment and that might thus have been due to acetazolamide, such as fatigue, ataxia, or gastrointestinal symptoms. Two patients withdrew from the trial due to the severity of side effects, on the recommendation of the clinical team (Supplementary Table 3).

### Exploratory endpoints

Five patients died during admission (2 in the acetazolamide arm vs. 3 in the standard-of-care arm). There was no statistical difference in the time to euvolaemia, the proportion of patients who achieved euvolaemia by the time of discharge, or the duration of admission between the treatment arms (Supplementary Table 4).

Similarly, there was no statistically significant difference in rehospitalisation or death at 30 or 180 days between arms (Supplementary Table 4).

### Per-protocol analysis

Results for the primary and secondary endpoints were the same on per-protocol analysis as they were for the intention-to-treat analysis (Supplementary Table 5).

## Discussion

Our pilot study suggests that oral acetazolamide 250 mg twice a day may protect against chloride loss during treatment with a high-dose IV loop diuretic but may not have much diuretic effect compared to a high-dose loop diuretic alone.

### Acetazolamide and diuresis

The ADVOR trial found that over three days, 500 mg of IV acetazolamide alongside low-dose IV furosemide compared to low-dose IV furosemide alone was associated with a greater diuresis (by 500 mL) and a greater natriuresis (by 98 mmol) after 48 h of treatment. The study was formally positive: acetazolamide was associated with a 46% greater chance of achieving euvolaemia after 48 h compared to placebo, with a number-needed-to-treat of 6 [8].

However, the study used IV acetazolamide, which is sometimes difficult to access, and only a low-dose loop diuretic as a comparator. Whether oral acetazolamide has any effect when added to high-dose IV furosemide is not known.

It is traditionally taught that mucosal oedema in the gastrointestinal tract reduces drug absorption in patients with congestion, but there is no difference in the diuretic or biochemical effect between IV and oral administration of adjunctive diuretics such as thiazide diuretics [13], or tolvaptan [12]. Acetazolamide appears to follow the same rule: although oral ACZ had no diuretic effect, we found that, like IV acetazolamide [13], it does affect serum and urine electrolytes, so it must be being absorbed [14].

Although our study was underpowered to detect modest differences, we found no statistically significant difference in diuresis. Similarly, the DEA-HF trial also found no additional diuresis with IV acetazolamide (one dose of 500 mg IV) in addition to high-dose IV furosemide (one dose of 250 mg IV) compared to IV furosemide alone in ambulatory patients with HF [15].

In the DOSE trial, high-dose furosemide (~ 260 mg per day) was superior to low-dose (~ 120 mg per day) for diuresis and improving symptoms of breathlessness [16]: the high-dose loop diuretic should be the comparator for any trial of combination diuretic therapy [17].

Most patients are admitted to hospital with HF with many litres of excess fluid1[1, 18], so although the difference in diuresis after 3 days found in ADVOR was statistically significant, it took a trial of 500 patients to detect it. It is unclear how a relatively small difference in diuresis may have contributed to the large effect size in the primary outcome of decongestion. Our data, taken with that of the DEA-HF trial, suggest that neither oral nor IV acetazolamide induces a clinically meaningful diuresis compared to high-dose furosemide alone [19]. However, both ADA-HF and DEA-HF recruited fewer than 10% of the number of patients randomised in ADVOR: ultimately, the effect of acetazolamide on diuresis is uncertain. The beneficial effects of acetazolamide, if they exist, may lie in its effect on chloride excretion.

### Acetazolamide and chloride

Hypochloraemia is a common side effect of loop diuretics [5, 6]. Post hoc analysis of the ADVOR trial found, as we did, preservation of serum chloride concentration in the acetazolamide arm and decreasing chloride concentrations in the standard-of-care arm. Patients who developed hypochloraemia in the ADVOR trial had a higher rate of re-admission and death at 5 years. However, ACZ treatment did not reduce the event rate, regardless of whether the patient was hypochloraemic or not12. The frequency of hypochloraemia both at baseline and after treatment was higher in ADA-HF than in ADVOR, probably because patients were more severely congested at baseline, and because we used higher doses of furosemide.

Acetazolamide might either prevent or correct hypochloraemia in patients with diuretic-induced hypochloraemia. There are two potential mechanisms by which acetazolamide reduces urinary chloride excretion: (1) by increasing urinary bicarbonate concentration, thereby increasing the negative electrochemical gradient from the urinary space into the renal medulla along which most chloride reabsorption occurs, and (2) by inhibiting the chloride-bicarbonate exchanger on the basolateral membrane of the proximal convoluted tubule reducing the movement of chloride out of the blood [7].

Whether hypochloraemia is a marker of severe disease or a mediator of adverse outcome in patients with HF is unknown. There are possible mechanisms to support the latter: myocyte volume and pH are regulated by chloride-dependent co-transporters, abnormalities of which may cause impaired myocardial contractility, or arrhythmia [3]. Increased chloride delivery to the macula densa suppresses renin secretion, thus, hypochloraemia may increase neurohormonal activation [3]. Hypochloraemia may also contribute to diuretic resistance [3], so maintaining chloride concentrations within the normal range may aid diuresis—this may, in part, explain the positive results of the ADVOR trial.

However, in our study, and in ADVOR, mortality and HF rehospitalisation were numerically higher with acetazolamide [8]. Our population was small, and the study was designed to capture patients with a degree of diuretic resistance; thus, the rates of adverse outcome should be interpreted with caution. Larger trials will be required to determine whether the beneficial effects of acetazolamide on chloride homeostasis in patients with advanced HF translate into outcome benefits beyond decongestion.

### A future for acetazolamide?

Prior to the advent of loop diuretics in the 1950 s, acetazolamide was one of the first-line treatments for fluid retention due to HF. Side effects were common: 14–38% of patients at doses > 1000 mg per day and 5–10% of patients at doses ≤ 500 mg per day reported side effects to the treatment [7]. Although there was no statistically significant increase in adverse events with acetazolamide in ADVOR, the rates of worsening renal function, hypokalaemia and hypotension were numerically greater in those who received acetazolamide [8]. The DEA-HF trial reported a greater rate of worsening renal function with acetazolamide [20]. Data on safety and tolerability of acetazolamide in modern studies have been inconsistently reported, and no trial has reported data on non-cardiac or non-renal side-effects [8–11]. We found that approximately 1 in 3 patients receiving acetazolamide experienced a non-cardiac or non-renal side effect.

Although our sample size was small, our results are consistent with other data. A meta-analysis of 42 trials including 1274 patients treated with oral acetazolamide for various conditions (mean age 44 years [considerably younger than most patients with HF, in whom the risk of side effects from the treatment is greater], mean acetazolamide dose 542 mg per day) found that symptoms such as paraesthesia or fatigue were very common, with numbers-needed-to-harm of 2 and 11 respectively. If a similar diuresis can be obtained with high-dose furosemide alone, it is unclear what role, other than to control serum chloride concentration with unclear clinical benefit, acetazolamide may play in managing patients with congestion due to HF [21].

### Limitations

As our pilot study was performed at a single centre, it may be argued that our results are not generalisable. However, our baseline characteristics are similar to those of patients recruited to other, larger trials of patients hospitalised with HF [7, 14, 15, 19].

We recruited patients at any time during hospitalisation as long as they were deemed to require at least 240 mg of IV furosemide per day. This entry criterion was chosen to select patients who had either not responded well to the initial low-dose treatment or were anticipated to have a degree of furosemide resistance on admission due to other clinical factors and thus require high-dose furosemide treatment from the outset. Both sets of patients may be considered to display “diuretic resistance” and, thus, be eligible for adjuvant therapies, such as acetazolamide. However, as a result, patients recruited to ADA-HF had received a median of 180 mg IV furosemide over a median period of 2 days prior to randomisation. By comparison, the ADVOR trial excluded patients who received > 80 mg of IV furosemide equivalents prior to randomisation. This is a key distinction between ADA-HF and ADVOR and may explain the more positive results of the latter.

Our sample size was small and we were unable to rule out a small difference in diuresis: the absolute difference between the arms after 96 h (492 mL) was similar to that seen in the ADVOR trial after 72 h (500 mL) but was not statistically significant. Our trial was of a similar size to DAPA-RESIST, which failed to demonstrate a clinically significant difference in diuresis between metolazone and dapagliflozin.

The absence of a placebo control means that firm conclusions regarding tolerability are difficult to draw. However, the side effects which led to withdrawal from the trial occurred only in the acetazolamide arm, and were due to side effects not known with furosemide (ataxia and paraesthesia). Side effects due to oral acetazolamide are common in other patient cohorts [20].

Approximately 1 in 4 patients in our trial were loop diuretic naïve prior to admission and thus it may be argued that neither high-dose furosemide treatment nor combination diuretic treatment would be appropriate. However, the median duration of hospitalisation prior to randomisation was 2 days, during which time patients had received a cumulative dose of 180 mg of IV furosemide yet were still deemed to require high-dose furosemide treatment due to inadequate initial response to treatment. It remains uncertain for whom high-dose loop diuretic or combination diuretic treatment is suitable.

Finally, like ADVOR, we excluded patients taking SGLT2I at baseline to ensure the only diuretic agents being tested were acetazolamide and furosemide. Although this may limit the generalisability of the results, it provides a clearer comparison between acetazolamide and furosemide versus furosemide alone.

## Conclusion

Oral acetazolamide at a dose of 250 mg twice daily does not induce a clinically meaningful diuresis when given in addition to 240 mg intravenous furosemide by continuous infusion. It was associated with a higher serum chloride concentration, but the side effects of acetazolamide were common.

## Supplementary Information

Below is the link to the electronic supplementary material.

ESM 1 Supplementary Fig. 1 Trial design and patient flow Legend: Abbreviations used: IV intravenous; SGLT2I sodium glucose co-transporter 2 inhibitor; SAE serious adverse event; ACZ acetazolamide; hr hour. Supplementary Fig. 2 Serum electrolytes and renal function Legend: A Change in serum sodium concentration; B change in serum potassium concentration; C change in serum bicarbonate concentration; D change in eGFR. Abbreviations used: eGFR estimated glomerular filtration rate.(DOCX 287 KB)

## Data Availability

Data will be made available upon reasonable request to the chief investigator.
