# Supplementary material for: ADA-HF: acetazolamide as a chloride sparing diuretic in patients admitted to hospital with heart failure
Source: Clin Res Cardiol. 2026 Apr 27;115(9):1675–85. doi: 10.1007/s00392-026-02915-5 (PMC13457235; doi:10.1007/s00392-026-02915-5)
Supplement: Supplementary file 1 [file 392_2026_2915_MOESM1_ESM.docx]

**Supplementary Table 1 – Urinary electrolytes**

|  | **Acetazolamide** | **Control** | **P** |
| --- | --- | --- | --- |
| **24-48 hours** | N =19 | N =21 |  |
| Total urine Sodium – mmol/24hr | 211 (116 – 276) | 191 (278 – 76) | 0.46 |
| Total urine chloride – mmol/24hr | 193 (124 – 249) | 195 (111 – 275) | 0.88 |
| Total urine potassium – mmol/24hr | 74 (48 – 102) | 53 (40 – 71) | 0.08 |
| Sodium concentration – mmol/L | 83 (69 – 112) | 79 (47 – 90) | 0.29 |
| Chloride concentration – mmol/L | 82 (62 – 106) | 84 (63 – 95) | 0.86 |
| Potassium concentration – mmol/L | 32 (21 – 37) | 24 (17 – 32) | 0.30 |
| **72-96 hours** | N = 11 | N = 11 |  |
| Total urine Sodium – mmol/24hr | 110 (67 – 172) | 122 (51 – 189) | 0.94 |
| Total urine chloride – mmol/24hr | 110 (66 – 151) | 134 (66 – 197) | 0.92 |
| Total urine potassium – mmol/24hr | **60 (44 – 87)** | **41 (28 – 51)** | **0.02** |
| Sodium concentration – mmol/L | 51 (47 – 85) | 67 (59 – 92) | 0.40 |
| Chloride concentration – mmol/L | 57 (43 – 84) | 68 (53 – 96) | 0.23 |
| Potassium concentration – mmol/L | 36 (24 37) | 27 (20 – 33) | 0.07 |

**Supplementary table 2 – Safety endpoints**

|  | **Control** | | **Acetazolamide** | | | **P** | |
| --- | --- | --- | --- | --- | --- | --- | --- |
| **Change in serum sodium concentration (mmol/L)** | | | | | | | |
|  | N |  | N |  |  | |  |
| Baseline value | 22 | 138 (135 – 143) | 21 | 138 (137 – 141) |  | |  |
| Change by Visit 5 - 96 hours | 19 | -1 (-5 – 1) | 17 | -2 (-3 – 0) | 0.68 | |  |
| *Hyponatraemia by 96 hours – N (%)^a^* | 19 | 6 (30) | 17 | 4 (22) | 0.72 | |  |
| **Change in serum potassium concentration (mmol/L)** | | | | | | | |
| Baseline value | 22 | 4.2 (3.6 – 4.5) | 21 | 3.8 (3.4 – 4.2) |  | |  |
| Change by Visit 5 - 96 hours | 19 | -0.1 (-0.7 – 0.2) | 17 | -0.4 (-0.5 – 0.3) | 0.84 | |  |
| *Hypokalaemia by 96 hours – N (%)^b^* | 19 | 3 (15) | 17 | 8 (47) | 0.07 | |  |
| **Change in serum bicarbonate concentration (mmol/L)** | | | | | | | |
| Baseline value | 22 | 30 (25 – 32) | 21 | 31 (26 – 33) |  | |  |
| Change by Visit 5 - 96 hours | 19 | 4 (2 – 7) | 17 | -3 (-5 – 3) | 0.05 | |  |
| *Metabolic alkalosis by 96 hours – N (%)^c^* | 19 | 10 (50) | 17 | 4 (22) | 0.17 | |  |
| *Metabolic acidosis by 96 hours – N (%)^d^* | 19 | 1 (5) | 17 | 1 (5) | 1.0 | |  |
| **Change in creatinine concentration (µmol/L)** | | | | | | | |
| Baseline value | 22 | 110 (86 – 143) | 21 | 104 (95 – 143) |  | |  |
| Change by Visit 5 - 96 hours | 19 | 18 (-2 – 50) | 17 | 22 (9 – 42) | 0.50 | |  |
| **Change in eGFR (ml/min/1.73m^2^)^e^** | | | | | | | |
| Baseline value | 22 | 54 (41 – 65) | 21 | 51 (39 – 67) |  | |  |
| Change by Visit 5 - 96 hours | 19 | -7 (-19 – 1) | 17 | -9 (-17 - -5) | 0.50 | |  |
| eGFR ≥25% increase from baseline by 96 hours – N (%) | 19 | 6 (32) | 17 | 6 (33) | 1.0 | |  |
| eGFR ≥50% of increase from baseline by 96 hours – N (%) | 19 | 2 (10) | 17 | 2 (12) | 1.0 | |  |

**Legend**

a - <135 mmol/L; b - <3.5 mmol/L; c - >32 mmol/L; d - <22 mmol/L; e - Calculated using Calculated using the CKD-EPI 2021 formula. Abbreviations used: N – number

**Supplementary Table 3 – Adverse events**

|  | **Control**  N = 23 | **Acetazolamide**  N = 23 | **P** |  |
| --- | --- | --- | --- | --- |
| Patients experiencing any adverse or serious adverse event – N (%) | 10 (43) | 16 (70) | 0.14 |  |
| Patients withdrawn from trial due to (S)AE prior to visit 5 ^a^ – N (%) | 0 | 4 (17) | 0.11 |  |
| **AE / SAE related to diuretic treatment or hospital admission** | | | | |
| Total number of (S)AEs – N | 29 | 51 | - |  |
| Worsening renal function^b^ – N (%) | 8 (35) | 8 (35) | 1.00 |  |
| Hyponatraemia^c^ – N (%) | 7 (30) | 5 (22) | 1.00 |  |
| Hypokalaemia^d^ – N (%) | 8 (35) | 15 (65) | 0.08 |  |
| Hypomagnesaemia^e^ – N (%) | 1 (4) | 0 | 1.00 |  |
| Hypotension^f^ – N (%) | 1 (4) | 5 (22) | 0.19 |  |
| *Symptomatic* | 0 | 2 |  |  |
| *Asymptomatic* | 1 | 3 |  |  |
| Gout – N (%) | 0 | 1 (4) | 1.0 |  |
| Muscle cramps – N (%) | 1 (4) | 1 (4) | 1.0 |  |
| Infection – N (%) | 1 (4) | 3 (13) | 0.61 |  |
| In-patient mortality – N (%) | 0 | 1 (4) | 1.00 |  |
| Escalation of loop diuretic therapy – N (%) | 0 | 0 | - |  |
| Renal replacement therapy – N (%) | 0 | 0 | - |  |
| Use of mechanical or inotropic support or IV vasodilator – N (%) | 0 | 0 | - |  |
| **Other notable side effects ^h^** | | | | |
| Hyperkalaemia – N (%) | 1 (4) | 0 | 0.38 |  |
| Abdominal pain / nausea / vomiting – N (%) | 1 (4) | 5 (21) | 0.64 |  |
| Drowsiness / parasthesiae / ataxia – N (%) | 0 | 4 (17) | 0.15 |  |
| Diaphoresis / flushing – N (%) | 0 | 1 (4) | 1.00 |  |
| Patients experiencing any side effect known to occur with acetazolamide ^h,i^ – N (%) | 0 | 8 (35) | 0.15 |  |
| Patients withdrawn from trial due to side effect– N (%) | 0 | 2 (9) | 0.52 |  |

**Legend**

a - One patient died between randomisation and visit 1, one patient developed a UTI and delirium and lacked capacity to continue, and two patients developed ataxia, paraesthesia and fatigue thought to be due to acetazolamide and stopped treatment (symptoms resolved after 24 hours off treatment); b – defined as >50% increase in serum creatinine concentration from baseline; c – defined as serum sodium <135 mmol/L; d - defined as any serum potassium <3.5 mmol/L; e – defined as serum magnesium <0.7 mmol/L; f – defined as systolic blood pressure < 80 mmHg; g – pain from catheter, constipation, high serum digoxin concentration (no clinical sequelae, no treatment needed), joint pain due to OA, urinary retention, hypothyroidism, and hyperkalaemia (serum potassium > 5.5 mmol/L) all N = 1; h – possibly due to acetazolamide, taken from the British National Formulary. I – one patient had two side effects possibly related to acetazolamide (abdominal pain and fatigue / ataxia) and was withdrawn from the trial.

**Supplementary table 4 - Outcomes**

|  | **Control**  N = 23 | **Acetazolamide**  N = 22 | **OR / HR (95% CI)** | **P** |  |
| --- | --- | --- | --- | --- | --- |
| In-patient mortality – N (%) | 3 (13) | 2 (9) | - | 1.00 |  |
| *Heart failure* | 2 | 0 |  |  |  |
| *Pneumonia* | 1 | 1 |  |  |  |
| *Sudden cardiac death* | 0 | 1 |  |  |  |
| Length of admission - days | 9 (7 – 14) | 14 (8 – 18) | - | 0.27 |  |
| Achieved euvolaemia – N (%)† | 174 (85) | 15 (71) | 0.44 (0.09 – 2.08) | 0.45 |  |
| Time to euvolaemia – days | 7 (5 – 10) | 7 (5 – 13) |  | 0.24 |  |
| **Outcomes at 30 days follow up** | | | | | |
| HF hospitalisation – N (%) | 1 (4) | 0 | - | - |  |
| All-cause hospitalisation – N (%) | 2 (9) | 2 (9) | 0.98 (0.06 – 15.59) | 0.99 |  |
| CV mortality – N (%) | 2 (9) | 2 (9) | 1.06 (0.15 – 7.54) | 0.95 |  |
| All-cause mortality – N (%) | 3 (13) | 3 (13) | 1.02 (0.21 – 5.04) | 0.96 |  |
| HF hospitalisation or CV mortality – N (%) | 2 (9) | 2 (9) | 1.06 (0.15 – 7.54) | 0.95 |  |
| HF hospitalisation or all-cause mortality | 3 (12) | 3 (12) | 1.06 (0.22 – 5.27) | 0.94 |  |
| All-cause hospitalisation or mortality | 4 (17) | 4 (17) | 1.04 (0.26 – 4.16) | 0.96 |  |
| **Outcomes at 180 days follow up** | | | | | |
| HF hospitalisation – N (%) | 4 (17) | 8 (35) | 1.03 (0.27 – 4.01) | 0.97 |  |
| All-cause hospitalisation – N (%) | 10 (44) | 12 (52) | 1.95 (0.73 – 5.21) | 0.18 |  |
| CV mortality – N (%) | 3 (13) | 3 (13) | 1.02 (0.20 – 5.17) | 0.97 |  |
| All-cause mortality – N (%) | 4 (17) | 5 (22) | 1.03 (0.25 – 4.18) | 0.97 |  |
| HF hospitalisation or CV mortality – N (%) | 6 (26) | 11 (50) | 2.02 (0.75 – 5.47) | 0.16 |  |
| HF hospitalisation or all-cause mortality – N (%) | 7 (30) | 12 (52) | 1.88 (0.74 – 4.79) | 0.18 |  |
| All-cause hospitalisation or mortality – N (%) | 13 (57) | 15 (65) | 1.16 (0.55 – 2.43) | 0.76 |  |

**Legend**

† - Odds ratio. Abbreviations used: CV - cardiovascular

**Supplementary table 5 – Primary and secondary endpoints per-protocol analysis**

|  | **Control**  **N = 21** | **Acetazolamide**  **N = 18** | **P** |
| --- | --- | --- | --- |
| Daily furosemide dose - mg | 240 (236 – 240) | 240 (238 – 240) | **-** |
| Cumulative furosemide dose (96 hours) – mg | 960 (948 – 960) | 955 (940 – 960) | **-** |
| **Primary endpoints** | | | |
| Cumulative fluid balance between 0 - 96 hours – mL | -3578 (-1219 - -5459) | -4070 (-2479 - -6073) | 0.76 |
| Median daily fluid balance (0 - 96 hours) – mL | -1010 (-192 - -1386) | -1033 (-555 - -1419) | 0.67 |
| Δ Chloride between 0 - 96 hours – mmol/L | -7 (-3 - -10) | 0 (2 – -2) | <0.001 |
| **Secondary endpoints** | | | |
| Cumulative urine output between 0 - 96 hours - mL | 8560 (5854 – 9275) | 8820 (7164 – 10211) | 0.51 |
| Median daily urine output (0-96 hours) - mL | 1885 (1408 – 2213) | 2258 (1480 – 2698) | 0.35 |
| Cumulative weight loss between 0 - 96 hours - kg | -5.6 (-1.5 - -7.7) | -3.7 (-2.5 - -6.6) | 0.81 |
| Δ Hull congestion score between 0 - 96 hours | -3 (-2 - -5) | -3 (-2 - -5) | 0.48 |
| Δ Modified ADVOR congestion score between 0 - 96 hours | -3 (-4 - -1) | -4 (-3 - -6) | 0.21 |
| Δ Likert score at rest between 0 - 96 hours | 0 (0 - -1) | -1 (0 - -1) | 0.13 |
| Δ Likert score on exertion between 0 - 96 hours | -1 (0 - -2) | -2 (-1 - -2) | 0.01 |
| Δ IVC diameter between 0 - 96 hours - cm† | -0.2 (-0.1 - -0.5) | -0.4 (-0.2 – -0.6) | 0.63 |
| Δ Sodium between 0 - 96 hours – mmol/L | -1 (1 - -5) | -2 (0 - -3) | 0.68 |
| Δ Potassium between 0 - 96 hours – mmol/L | -0.1 (0.2 -0.7) | -0.4 (0.3 - -0.5) | 0.84 |
| Δ Bicarbonate between 0 - 96 hours – mmol/L | 4 (2 - 7) | -3 (3 - -5) | 0.05 |
| Δ Creatinine between 0 - 96 hours – µmol/L | 18 (-2 – 50) | 22 (9 – 42) | 0.50 |
| Δ eGFR between 0 - 96 hours – ml/min/1.73m^2^ | -7 (1 - -19) | -9 (-5 - -17) | 0.50 |

**Legend**

† - Patients with IVC diameter measurements at baseline and 96 hours N = 16 in the control arm and N = 12 in the acetazolamide arm. Abbreviations used: IVC – inferior vena cava; eGFR – estimated glomerular filtration rate.

Supplementary figure 1

Supplementary figure 2
